# Brain activation generated by periodontal initial treatment

**DOI:** 10.1016/j.jds.2025.04.005

**Published:** 2025-04-21

**Authors:** Kosuke Muraoka, Masafumi Oda, Kenichi Yoshino, Tatsurou Tanaka, Masaki Morishita, Taiji Nakamura, Shino Yamaguchi, Kazuo Sonoki, Yasuhiro Morimoto, Keisuke Nakashima, Shuji Awano

**Affiliations:** a Division of Clinical Education Development and Research, Faculty of Dentistry, Kyushu Dental University, Kitakyushu, Japan; b Division of Oral and Maxillofacial Radiology, Kyushu Dental University, Kitakyushu, Japan; c Section of Primary Dental Education, Kyushu Dental University, Kitakyushu, Japan; d Graduate School of Medical and Dental Sciences, Department of Maxillofacial Radiology, Kagoshima University, Kagoshima, Japan; e Division of Periodontology, Kyushu Dental University, Kitakyushu, Japan; f School of Oral Health, Kyushu Dental University, Kitakyushu, Japan; g Sonoki Internal Medicine Clinic, Fukuoka, Japan

**Keywords:** Brain activation, Calculation task, fMRI, Language task, Periodontal initial treatment

## Abstract

**Background/purpose:**

There is a close relationship between the dental field and the cognitive function of the brain. The aim of this research was to examine the effect of periodontal initial treatment on brain functional activity.

**Materials and methods:**

The subjects were 16 patients with periodontitis. After informed consent was obtained, periodontal initial treatment included brushing instruction, scaling and root planing. No occlusal adjustments were conducted. Periodontal examination and functional magnetic resonance imaging (fMRI) were performed at the initial visits and subsequent reassessments.

**Results:**

Periodontal tissue improved significantly after periodontal initial treatment. From fMRI testing, during both the language and calculation task, brain activity in the dorsolateral prefrontal cortex and Wernicke's area were observed to have increased compared to the initial visit.

**Conclusion:**

Periodontal disease causes functional impairment of working memory and selective attention. These findings suggested that the periodontal initial treatment aid in restoring brain function.

## Introduction

The world is fast becoming an aging society, and therefore symptoms and cases of dementia are becoming a major issue. Most cases of dementia are difficult to treat, and no therapeutic drugs have been developed to date. Cognitive functions include various aspects of intellectual behavior such as memory, language, and problem-solving. Systemic factors related to the decline in cognitive function and the onset of dementia include malnutrition^1^ and lifestyle-related diseases.^2^ Additionally, there are also reports on the relationship between cognitive decline, dementia onset and the oral cavity with regard to the chronic inflammation of periodontal tissue^3^ and tooth loss.^4^

Good oral health may contribute to the prevention of cognitive decline, so dental professionals need to promote the prevention, early detection, and early treatment of oral diseases. Therefore, oral care is important for dementia prevention. Kikutani et al.^5^ reported that subjects who received oral care had significantly less cognitive decline than those who did not receive oral care. Periodontal initial therapy in patients with chronic periodontitis improved periodontal tissue and increased brain activity during bitewing movements.^6^ In other words, there is a relationship between cognitive decline and oral health.

One method of evaluating brain function is functional magnetic resonance imaging (fMRI). fMRI is a testing method that can objectively extract brain activation sites associated with neural activity. In recent years, fMRI has been clinically applied because it is non-invasive, has excellent temporal and spatial resolution, and does not involve radiation exposure. Therefore, we theorized that periodontal initial treatment would improve the condition of the periodontal tissue and consequently activate brain function.

The focus of this study was to research whether periodontal initial treatment affects brain function activation.

## Materials and methods

### Study design

This research was accepted by the Kyushu Dental University Ethics Committee (No. 11–39) and was undertaken in accordance with the Helsinki Declaration of 1975, as revised in 2013.

This study centered on 16 subjects (5 males and 11 females) with the average age of 61.7 ± 8.3 years. All patients had previously been diagnosed with periodontitis, at the Kyushu Dental University Hospital. The average number of teeth of the subjects were 22.9 ± 4.6.

Six of these patients had also developed systemic diseases. Hypertension was evident in three of these. Type 2 diabetes mellitus in two patients, hyperlipidemia and rheumatium in a single patient. Testing of the subjects had proven that they were all under the standard blood test values.

### Examination

The probing pocket depth (PPD), the clinical attachment level (CAL), and the incidence of bleeding upon probing of gingiva the (%BOP (+)) were evaluated at the initial visit and the following reevaluation visit.

MRI scans were conducted using a 1.5-T full-body MR system (EXCELART Vantage™ Powered by Atlas; Toshiba, Tokyo, Japan) at the Dental Radiology Department of Kyushu Dental University Hospital. Brain activity sites were measured using fMRI, following the noted procedure.^7^ Each subject was escorted into the MRI room, equipped with ear protection to mitigate noise during the MRI examination, and provided with instructions. The subject was then positioned supine for imaging. To impede any mobility of the subject's head, a pad and belt were implemented to secure the forehead. Directions were provided from the control room for the patient to perform language and calculation tasks. Two distinct types of these tasks were utilized, and the subject alternated between performing the specific cognitive tasks and the control tasks for 30 s each, repeating this cycle three times.

These tasks performed during fMRI were conducted according to Nakashima et al.^8^ In the language task, the patient was asked to think of as many words as possible using Japanese phonics. The operator in the control room spoke the Japanese phonetic sounds “Aka (Red) et al.”, “Kasa (Umbrella) et al.”, “Sakura (Cherry blossom) et al.” and so on, which were subsequently relayed to the MRI room. The subject was then asked to provide words beginning with ‘a’, ‘ka’, or ‘sa’ as a phonetic cue. In the control task, ‘a, i, u, e, o et al.’ is the repetition of the sequence of Japanese vowels ‘a, i, u, e, o et al.’. After being given a phonetic cue for the Japanese “a” column corresponding to the former language task, the patient repeated the Japanese “a”, “ka”, and “sa” columns (a, i, u, e, o et al.).

In the calculation task, the audio cues “400”, “300”, and “200”, were provided as in the language task, and the subject was asked to subtract 7 from “400 counting down for 30 s”, “300 counting down for 30 s”, and “200 counting down for 30 s” in that order, respectively. In the control task, the subject was required to repeatedly subtract 1 from 100.

During the specific cognitive tasks and control task at the time of imaging, the patient was instructed to close their eyes, to remain silent, and not be in a state of occlusion.

### Periodontal treatment

After the subject's written, informed consent was obtained, periodontal initial treatment was performed. The periodontal initial treatment included tooth brush instruction, scaling and root planing. Periodontal treatments were given once every one to two weeks. The duration of the periodontal initial treatment period was 158.0 ± 57.3days.

### Statistical analysis

After all parameters were examined for normal distribution, all parameters were analyzed by paired t-test. JMP8.0.2® (SAS Ins, Cary, NC, USA) was used for statistical analysis.

The fMRI data was analyzed using SPM 8 (London, UK), and the analysis was carried out with Matlab 7.11 (Mathworks, Sherborn, MA, USA). The images obtained from the functional scans were normalized to conform to the standard brain template provided by the Montreal Neurological Institute. The significance of the voxels and activation areas, characterized by their peak heights, was determined using the t-test (***P*** < 0.001).

## Results

The fMRI and periodontal tissue data for 16 patients (5 males and 11 females) with periodontal treatment are shown below.

### Change of brain activation sites identified by fMRI

**Figure 1:**
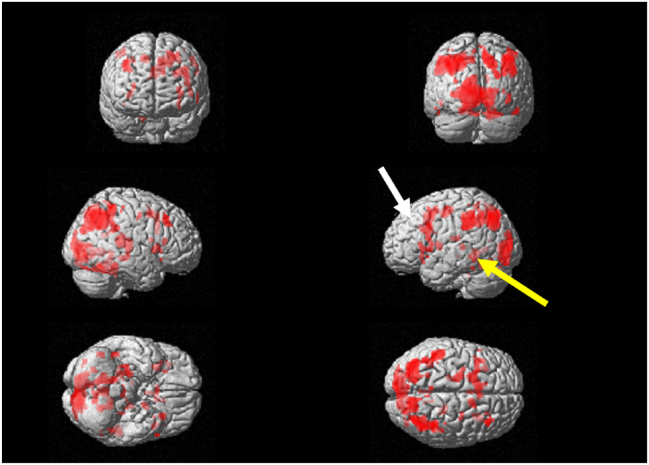
Surface projection of statistical parametric maps superimposed onto a standard Montreal Neurological Institute brain template (***P*** < 10^−3^) during the language task at the initial visit. The dorsolateral prefrontal cortex (white arrow) and the Wernicke's area (yellow arrow) were activated. (For interpretation of the references to color in this figure legend, the reader is referred to the Web version of this article.)

**Figure 2:**
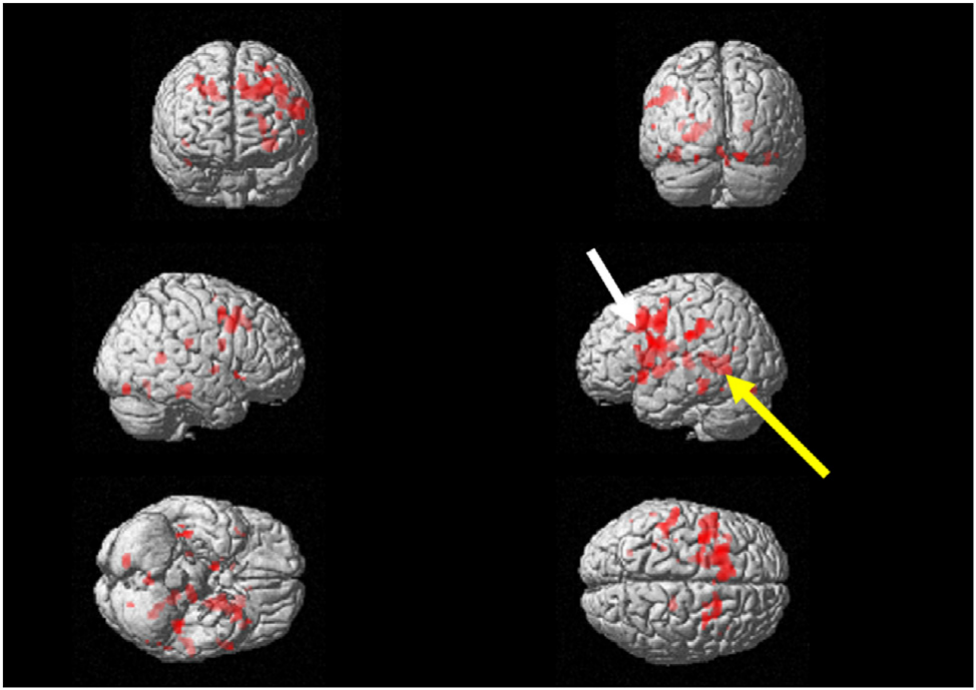
Surface projection of statistical parametric maps superimposed onto a standard Montreal Neurological Institute brain template (***P*** < 10^−3^) during the language task at the reevaluation visit. The dorsolateral prefrontal cortex (white arrow) and the Wernicke's area (yellow arrow) were activated. (For interpretation of the references to color in this figure legend, the reader is referred to the Web version of this article.)

In the language task, significant brain activity was noted in the left dorsolateral prefrontal cortex at the initial visit (Fig. 1). Furthermore, there was a slight increase observed in brain activity within the Wernicke's area. The T-values for the left dorsolateral prefrontal cortex and the left Wernicke's area were recorded as 4.27 and 4.47, respectively (Table 1). Brain activity in the left dorsolateral prefrontal cortex and Wernicke's area was observed to have increased compared to the first visit (Fig. 2). The T-values for the left dorsolateral prefrontal cortex and the Wernicke's area were recorded as 6.06 and 5.38, respectively (Table 1).

**Table 1:** T-values of the dorsolateral prefrontal cortex and Wernicke's area at the initial and reevaluation visits.

	Initial visit				Reevaluation visit			
	Coordinates				Coordinates			
	T-value	x	y	z	T-value	x	y	z
Language task								
Dorsolateral prefrontal cortex	4.27	−40	20	28	6.06	−34	18	36
Wernicke's area	4.47	−28	−32	2	5.38	−28	−34	8
Calculation task								
Dorsolateral prefrontal cortex	5.34	−40	10	28	7.39	−42	8	20
Wernicke's area	5.57	−34	−38	10	6.18	−32	−30	−4

**Figure 3:**
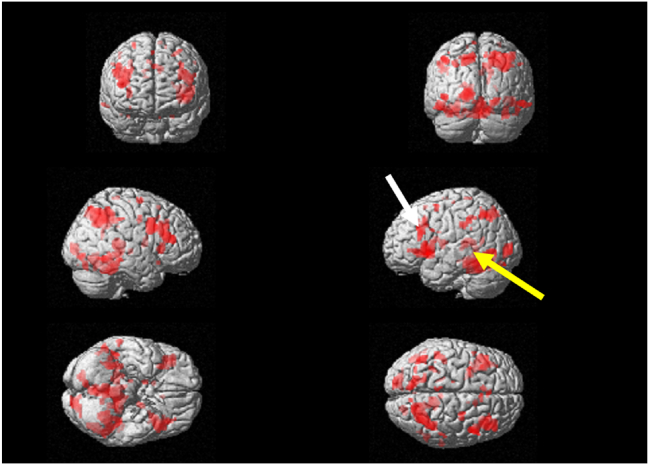
Surface projection of statistical parametric maps superimposed onto a standard Montreal Neurological Institute brain template (***P*** < 10^−3^) during the calculation task at the initial visit. The dorsolateral prefrontal cortex (white arrow) and the Wernicke's area (yellow arrow) were activated. (For interpretation of the references to color in this figure legend, the reader is referred to the Web version of this article.)

**Figure 4:**
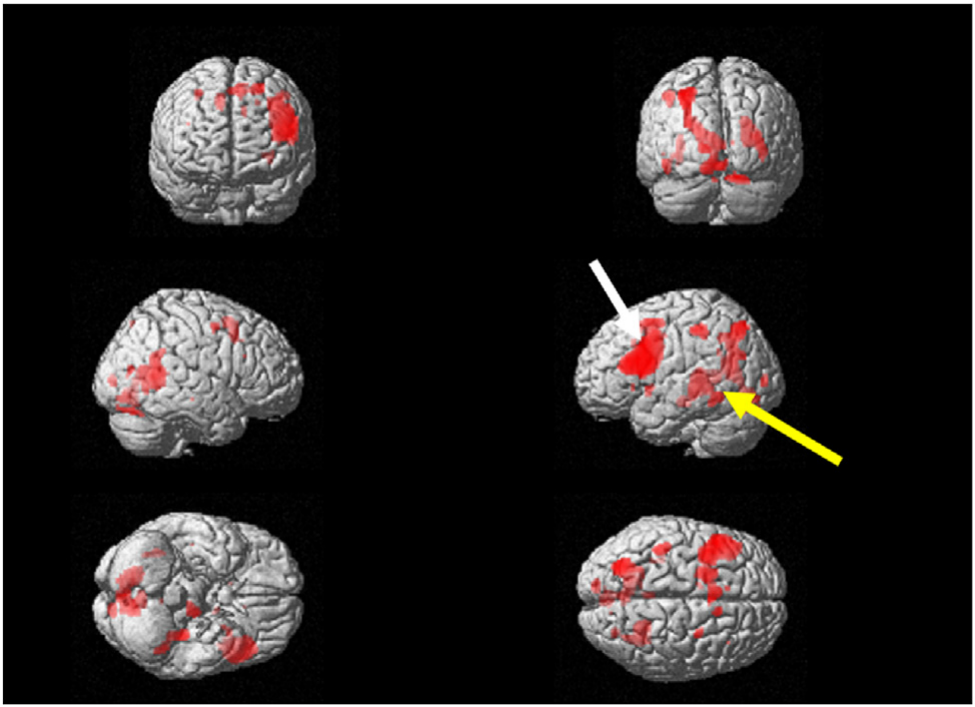
Surface projection of statistical parametric maps superimposed onto a standard Montreal Neurological Institute brain template (***P*** < 10^−3^) during the calculation task at the reevaluation visit. The dorsolateral prefrontal cortex (white arrow) and the Wernicke's (yellow arrow) were activated. (For interpretation of the references to color in this figure legend, the reader is referred to the Web version of this article.)

In the calculation task, a significant increase in brain activity from the first visit was observed in the left dorsolateral prefrontal cortex, and a significant increase was also noted in the left Wernicke's area (Fig. 3). The T-values for the left dorsolateral prefrontal cortex and the Wernicke's area were recorded as 5.34 and 5.57, respectively (Table 1). Brain activity was observed to have increased in comparison to the first visit in the left dorsolateral prefrontal cortex and the left Wernicke's area (Fig. 4). The T-values for the left dorsolateral prefrontal cortex and the Wernicke's area were recorded as 7.39 and 6.18, respectively (Table 1), higher than those at the first visit.

### Periodontal tissue examination

Table 2 shows the comparison of periodontal tissue at the initial visit and the reevaluation. Periodontal treatment generated significant betterment in the periodontal tissues in the average PPD, the average CAL and the average %BOP (+).

**Table 2:** Comparison of periodontal tissue and occlusal force at the initial visit and the reevaluation.

	Ave PPD (mm)	Ave CAL (mm)	%BOP (+)
Initial	3.3 ± 0.9	3.7 ± 1.0	39.4 ± 22.9
Reevaluation	2.6 ± 0.7	3.1 ± 1.1	18.5 ± 10.4
*P*	***0.0009***	***0.0031***	***0.0004***
			(n = 16)

Ave PPD: Probing pocket depth.

Ave CAL: Clinical attachment level.

%BOP(+): Bleeding on probing as a percentage of the measured area.

Values are presented as mean ± standard deviation.

## Discussion

Cognitive decline is a difficult condition to treat. Therefore, the need to prevent the onset of cognitive decline, as well as the early identification of this deterioration is of great importance.

In diseases associated with cognitive decline, metabolic syndrome is recognised as a hazard for the gradual evolution of cognitive deterioration,^9^ and hypertension in the metabolic syndrome has been reported to cause cognitive decline.^10^ Furthermore, after the onset of cognitive decline, weight loss has been reported as an additional risk factor for cognitive decline.^11^ From these reports, it was thought that cognitive decline was only related to an individual's overall physical condition.

There is normal cognitive function as a result of normal blood pressure with antihypertensive therapy.^12^ In other words, the hypertensive subjects in this study also had no problems with brain function regarding antihypertensive therapy.

However, in recent years, reports have emerged linking the oral region to cognitive decline. It has been reported that individuals with fewer lost teeth have a lower risk of cognitive decline.^13^ Additionally, tooth loss has been reported to affect the proteins that cause Alzheimer's disease.^14^ Periodontal disease is influenced by periodontal pathogenic bacteria known as the red complex. Among them, Porphyromonas gingivalis is considered to be the most signficant in periodontal disease. Porphyromonas gingivalis has been frequently found in the brain tissue of subjects who died of Alzheimer's disease, but not in healthy individuals.^15^ This result suggests the possibility of some pathway. Zeng et al.^16^ reported that Porphyromonas gingivalis infects the entire body, causing amyloid β to be taken up into the brain in peripheral inflammatory tissues, inducing memory impairment. In other words, periodontal pathogenic bacteria may spread to the brain and cause cognitive decline. And Nobel et al.^17^ also reported that periodontal disease caused functional changes in both memory and cognition. In conclusion, we believe that the relationship between recognition disorders and periodontal disease are related.

However, there have been no studies on the impact of periodontal treatment on higher brain functions. Therefore, we theorized that periodontal treatment would improve periodontal tissues and consequently improve higher brain functions.

In the both language and calculation task, brain activity in the dorsolateral prefrontal cortex and Wernicke's areas were observed to have increased at the reevaluation visit when compared with that in initial visit. This study suggests that periodontal initial treatment has a significant effect on the activity of the dorsolateral prefrontal cortex and Wernicke's area. In order for the human brain to operate effectively, in regards to cognitive function, future planning and behavioral choices, the dorsolateral prefrontal cortex, and Wernicke's area are vital.

The dorsolateral prefrontal cortex and Wernicke's area play important roles in memory and calculation tasks. Periodontal disease could interfere with the function of the dorsolateral prefrontal cortex and Wernicke's area. Thus, periodontal initial treatment may be able to eliminate these negative effects. These results suggest that periodontal treatment leads to the increase of brain activity.

Cognitive decline is thought to be influenced by periodontal disease and tooth loss.^3^^,^^4^ Oue et al.^18^ demonstrated a decline in cognitive function after the extraction of molars in mice. However, they reported that amyloid β protein in Alzheimer's disease was not affected.

In this study, without extracting teeth, we performed only periodontal initial treatment and observed the activation of brain functions. Therefore, periodontal initial treatment may reduce the impairment of brain activity. In the future, the number of samples is also small and will be increased to evaluate the brain function in more detail.

## Declaration of competing interest

The authors have no conflicts of interest relevant to this article.
